# Lactate Enhances Non-Homologous End Joining Repair and Chemoresistance Through Facilitating XRCC4–LIG4 Complex Assembly in Ovarian Cancer

**DOI:** 10.3390/biomedicines13122949

**Published:** 2025-11-30

**Authors:** Jingyi Lu, Jiayu Zhu, Huanxiao Zhang, Zhou Zhou, Haoyuan Li, Cuimiao Zheng, Xi Huang, Siqi Chen, Chaoyun Pan, Jie Li, Hao Tan

**Affiliations:** 1 Department of Obstetrics and Gynecology, The First Affiliated Hospital, Sun Yat-sen University, Guangzhou 510080, China; lujy257@mail.sysu.edu.cn (J.L.); huanxiao.zhang@163.com (H.Z.);; 2 Department of Biochemistry and Molecular Biology, Zhongshan School of Medicine, Sun Yat-sen University, Guangzhou 510080, China

**Keywords:** lactate, ovarian cancer, NHEJ, chemoresistance

## Abstract

**Background**: Non-homologous end joining (NHEJ) is a crucial pathway for repairing DNA double-strand breaks and a key contributor to chemoresistance in cancer. The assembly of the DNA Ligase IV (LIG4)–XRCC4 complex is essential for NHEJ fidelity, however, the regulatory mechanisms governing this complex in cancer remain poorly understood. This study aims to investigate whether and how lactate, a key metabolic byproduct of the Warburg effect, regulates the XRCC4–LIG4 complex and influences chemoresistance. **Methods**: The functional role of lactate in NHEJ was assessed using DNA repair reporter assays in ovarian cancer cells. Protein–protein interactions were examined through co-immunoprecipitation and pull-down assays. The molecular mechanism of lactate’s action was delineated using a combination of site-directed mutagenesis, in vitro binding assays, and molecular docking. Finally, the physiological relevance of lactate-mediated NHEJ was validated in a preclinical ovarian cancer mouse model treated with cisplatin. **Results**: We demonstrated that lactate enhances NHEJ repair efficiency and confers resistance to DNA-damaging chemotherapeutics. Mechanistically, lactate directly binds to XRCC4 at key residues, including Y66, E55, and S110, thereby strengthening the XRCC4–LIG4 association. This interaction is independent of protein lactylation. In vivo studies confirmed that lactate-driven NHEJ promotes chemoresistance in ovarian cancer. **Conclusions**: Our findings reveal lactate as a novel metabolic regulator of the NHEJ pathway by directly allosterically modulating the XRCC4–LIG4 complex. This work establishes a direct molecular link between the Warburg effect and DNA repair-driven chemoresistance, offering new insights into potential therapeutic strategies for ovarian cancer.

## 1. Introduction

Ovarian cancer remains one of the most lethal malignancies in women, ranking fifth in cancer-related mortality and surpassing all other gynecologic cancers in fatality rates [1]. For decades, platinum-based chemotherapy has served as the cornerstone of first-line treatment for ovarian cancer [2,3]. Although initially effective, the chemotherapy regimen frequently leads to the development of severe drug resistance in later stages, ultimately contributing to disease recurrence and poor patient outcomes [4,5]. Deciphering the molecular mechanisms underlying platinum resistance is therefore critical for improving therapeutic strategies in ovarian cancer [6].

Extensive research has focused on DNA damage repair, especially double strand breaks (DSBs), to elucidate the basis of platinum resistance [7]. There are two main pathways responsible for repairing DSBs as follows: non-homologous end joining (NHEJ) and homologous recombination (HR) [8]. Notably, ovarian cancer cells exhibiting homologous recombination deficiency (HRD) display heightened sensitivity to both PARP inhibitors and platinum-based drugs [9,10]. Conversely, the restoration of homologous recombination (HR) confers significant resistance to platinum agents, highlighting HR’s pivotal role in cisplatin resistance [11]. Unlike HR, which relies on an undamaged DNA template for high-fidelity repair, NHEJ operates independently of homology, rapidly ligating broken DNA ends with lower accuracy [12,13]. Despite its reduced precision, NHEJ is active throughout the cell cycle, whereas HR is predominantly restricted to the S-phase [14]. Given these differences, NHEJ may serve as a complementary mechanism to HR, ensuring timely DNA repair across all cell cycle phases, albeit at the cost of fidelity. Consequently, targeting NHEJ may present a promising therapeutic avenue for overcoming platinum resistance.

The Warburg effect, or aerobic glycolysis, is a well-established metabolic hallmark of cancer, characterized by excessive lactate production despite oxygen availability [15,16,17]. Lactate, generated from pyruvate via lactate dehydrogenase (LDH), plays multifaceted roles in tumor progression, including promoting proliferation, metastasis, and immune evasion [18,19]. Beyond its metabolic functions, emerging evidence suggests that lactate also mediates non-canonical regulatory mechanisms. For instance, recent studies have identified lactylation—a novel post-translational modification (PTM) of histone lysine residues—as an epigenetic regulator of gene expression [20]. Additionally, lactate has been shown to modulate HR repair through the lactylation of MRE11 [21] or NBS1 [22], as well as influence cell cycle progression by inhibiting SENP1 and remodeling the anaphase-promoting complex [23]. While lactate has been preliminarily linked to the regulation of NHEJ and chemoresistance and Dr. Wagner’s team were indeed pioneering in linking lactate to NHEJ-mediated chemoresistance, specifically through the regulation of DNA-PK and LIG4 [24], a direct and mechanistic understanding of how lactate influences the core NHEJ machinery remains elusive. Specifically, although their work implicated DNA-PK and LIG4, it did not establish whether lactate exerts its effect through a direct physical interaction with the core ligation complex components or through indirect, upstream signaling. The fundamental question of whether lactate can directly bind to and allosterically regulate the pivotal XRCC4–LIG4 complex—the essential unit responsible for the final DNA ligation step in NHEJ—and thereby dictate repair efficiency and chemoresistance, is still unresolved. Our study was designed to directly address this critical gap in knowledge.

In this study, we reveal that lactate enhances NHEJ-mediated DNA repair and confers chemoresistance in ovarian cancer by directly facilitating the interaction between XRCC4 and LIG4, the key event in NHEJ machinery assembly [25]. Mechanistically, lactate does not induce the lactylation of XRCC4 or LIG4 but instead binds to XRCC4 at Arg453, stabilizing the XRCC4–LIG4 complex and augmenting NHEJ efficiency. Using a preclinical mouse model, we further demonstrate that lactate-driven NHEJ activation promotes chemotherapy resistance in ovarian cancer. These findings establish lactate as a metabolic regulator of NHEJ and uncover a direct connection between the Warburg effect and chemoresistance in ovarian cancer.

## 2. Materials and Methods

**Cell lines:** A2780 (RPMI-1640, 10% FBS, 1% penicillin-streptomycin), SKOV3 (McCoy’s 5a, 10% FBS, 1% penicillin-streptomycin), OVCAR-433 (DMEM, 10% FBS), 293T (DMEM, 10% FBS), ID8 (DMEM, 5% FBS, 1% insulin-transferrin-selenium-ethanolamine (ITS-X), 1% penicillin-streptomycin). All commercial cell lines were authenticated by STR profiling. Cell lines were obtained from the American Type Culture Collection (ATCC, Manassas, VA, USA).

**Animal studies:** C57BL/6 mice (C57BL/6NCrl, female, 6 weeks old, Charles River, Beijing, China). The mice were maintained under optimal housing conditions to ensure well-being and minimize stress. The housing environment featured a controlled 14 h light/10 h dark cycle, with temperature maintained at 18–23 °C and humidity at 40–60%. For the ID8 intraperitoneal model, humane endpoints included: a 20% increase in abdominal circumference, palpable tumor mass, or hemorrhagic ascites upon intraperitoneal injection.

**Reagents and Kits:** All key reagents used in this study are listed in Supplementary Table S1. For cell treatments: L-lactate was used at concentrations of 0, 10, 20 mM, dissolved in phosphate-buffered saline (PBS). The LDH inhibitor (LDHi, [sodium oxamate]) was used at concentrations of [LDHi Concentrations 0, 10, 20 mM], dissolved in dimethyl sulfoxide (DMSO). The standard treatment duration was 24 h. The final concentration of DMSO in the culture medium was kept below 0.1% (*v*/*v*). Cisplatin: A concentration gradient of 0, 2.5, 5, and 10 µM was used. Etoposide: A concentration gradient of 1, 5, and 10 µM was used. Lactate: Concentration gradients of 0, 10, and 20 mM were used. LDHi: Concentration gradients of 0, 10, and 20 mM were used.

**Measurement of Lactate levels:** Cells seeded in six-well plates (triplicate wells) were treated with L-lactate at a concentration of 10 mM or 20 mM for 24 h, or LDHi at a concentration of 10 mM or 20 mM for 24 h. The intracellular lactate level was measured by using the lactate Colorimetric/Fluorometric assay kit (Abcam ab65331, Cambridge, MA, USA) according to the manufacturer’s protocol. L-lactate was dissolved in phosphate-buffered saline (PBS), and the LDH inhibitor (LDHi) was dissolved in dimethyl sulfoxide (DMSO).

**NHEJ reporter assay:** DNA double-strand break repair efficiency was assessed using cNHEJ reporter systems. Cells seeded in six-well plates (triplicate wells) were first treated with L-lactate at a concentration of 10 mM or 20 mM for 24 h, or LDHi at a concentration of 10 mM for 24 h, then transfected with 500 ng cNHEJ reporter plasmid, 100 ng mCherry plasmid (transfection control), 500 ng I-SceI plasmid (to induce DSBs), and 3 μL Lipofectamine 2000. After 48 h, cells were analyzed by flow cytometry for the GFP+ (repaired) and mCherry+ populations. DNA repair efficiency by comparing the number of GFP-positive cells. The percentage of mCherry-positive cells in experimental samples serves as the normalization control of transfection efficiency. Data are presented as the percentage of GFP-positive cells normalized to the percentage of mCherry-positive cells in each sample and are shown as the mean ± SD from 3 biological replicates.

**Cell cycle profile:** Cell Cycle Analysis Kit (no. C1052; Beyotime, Shanghai, China) was used in cell cycle assay. Cells seeded in six-well plates (triplicate wells) were treated with L-lactate at a concentration of 10 mM or 20 mM for 24 h, or LDHi at a concentration of 10 mM or 20 mM for 24 h, then harvested and fixed in 70% ethanol for 2 h at 4 °C, and then stained with a solution containing propidium iodide (0.05 mg/mL), RNase A (1 mg/mL), and 0.3% Triton X-100 in the dark for 30 min. The percentage of cells in different phases of the cell cycle was examined by measuring the DNA content (propidium iodide intensity) with a flow cytometer (Beckman Coulter, Brea, CA, USA), and populations of G1, S, and G2/M phase cells were determined with the ModFIT software, V3.3. Each experiment was repeated three independent times.

**Immunoprecipitation:** For Flag-tagged or Myc-tagged protein immunoprecipitation, anti-Flag M2 magnetic beads (Sigma, A2220, St. Louis, MO, USA) or BeyoMag™ Anti-Myc Magnetic Beads (Beyotime, P2183S) were used according to the manufacturer’s instructions. For each IP, 2 million cells were lysed in 1% NP-40 buffer [50 mM Tris-HCl (pH 7.5), 150 mM NaCl, 1% NP-40] containing protease inhibitor cocktail (Roche, Basel, Switzerland). Negative controls used equivalent lysate volumes from untransfected cells. Beads were equilibrated, incubated with lysates overnight at 4 °C with rotation, then collected magnetically. After three TBS washes, bound proteins were eluted with 100 μL 3X Flag peptide (150 ng/μL in TBS) or Myc Peptide for downstream Western blot analysis.

**Western blot:** For cellular protein analysis, cells were lysed in buffer containing 50 mM Tris-HCl (pH 7.5), 150 mM NaCl, 1% NP-40, and 0.5% sodium deoxycholate supplemented with protease inhibitor cocktail (Roche). Total protein (30 μg) was resolved by 10% SDS-PAGE and transferred to nitrocellulose membranes (Bio-Rad, Hercules, CA, USA). Membranes were probed with primary antibodies overnight at 4 °C, followed by TBST washes and incubation with HRP-conjugated secondary antibodies (1 h, RT). Protein signals were detected using enhanced chemiluminescence and quantified with ImageJ software (version 1.8.0), bundled with 64-bit Java 1.8.0 172

**Cell viability assay:** Cell viability was assessed using a Trypan blue exclusion kit (Beyotime, #C0011) according to the manufacturer’s protocol. Following the indicated treatment, live (unstained) and dead (blue-stained) cells were automatically detected and quantified using an AI-powered cell counter (Countess 3, Thermo Fisher Scientific, Waltham, MA, USA).

**The CCK-8 cell viability assays:** This assay was assessed under longer treatment durations (72 h) and across a range of cisplatin concentrations (0, 2.5, 5, and 10 µM).

**Cellular thermal shift assay:** Flag-XRCC4 or Myc-LIG4 overexpressing cells (1 × 10^6^) were harvested 48 h post-transfection and resuspended in 1 mL PBS containing protease inhibitors. Cell lysates were prepared through three freeze–thaw cycles using liquid nitrogen, followed by centrifugation (20,000× *g*, 20 min, 4 °C). Cleared supernatants were aliquoted (50 μL/tube), incubated with L-lactate for 30 min at RT, and subjected to temperature gradient treatment (3 min per temperature) in a PCR cycler. Heat-treated samples were immediately processed for Western blot analysis.

**Molecular docking:** The three-dimensional structure of L-lactate (Compound CID: 5460161) was retrieved from the PubChem database in SDF format. The ligand structure was then imported into Molecular Operating Environment (MOE) software, version 2019.0102. and subjected to energy minimization using the MMFF94 force field with an RMS gradient convergence criterion of 0.001, after which it was saved in MOE format. The crystal structure of XRCC4 protein (PDB ID: 1FU1) or LIG4 protein (PDB ID: 3W5O) was obtained from the Protein Data Bank and prepared using MOE’s QuickPrep module. This preparation involved protonation of ionizable residues, hydrogen atom addition, active center tether assignment, positional optimization of distal atoms, and energy minimization to obtain the most stable conformation. Docking simulations were performed using the dimer interface of XRCC4 or LIG4 as the binding site. The London dG scoring function was employed to evaluate binding free energy, while GBVI/WSA dG calculations provided binding energy (ΔG) estimates. From 300 generated docking poses, the top five conformations with the lowest ΔG values were selected for detailed analysis. Molecular interactions were visualized and analyzed using PyMOL version 2.3.0.

**ID8 ovarian cancer chemotherapy model:** GFP-luciferase-expressing murine ID8 ovarian cancer cells were enriched by GFP sorting and expanded in culture. After overexpressing Flag XRCC4 WT or Y66F mutant, a total of 5 × 10^6^ luciferase-positive ID8 cells were intraperitoneally injected into 6-week-old female C57BL/6 mice (Charles River, Wilmington, MA, USA). Cisplatin (5 mg/kg, i.p., twice weekly) and LDHi (750 mg/kg, i.p., once per day) were administered during the specified treatment timeline. Tumor progression was monitored weekly by in vivo bioluminescence imaging (Living Image software, PerkinElmer, version 4.1).

**Statistical analysis:** GraphPad Prism 8 was used for statistical analysis and graphical presentation. Data are shown as the mean ± SD or SEM (as specified in figure legends), with no exclusions. Significance was determined using the two-tailed Student’s *t*-test or one-way/two-way ANOVA with Bonferroni post hoc testing (as noted in legends). Analyses assumed normal distribution, homogeneity of variance, and comparable variance between groups (*p* < 0.05 considered significant). For in vivo experiments, sample sizes were based on preliminary data demonstrating detectable effects (no formal power calculation), and no statistical method was used to predetermine sample size; blinding was not performed.

## 3. Results

### 3.1. Lactate Promotes NHEJ Repair in Ovarian Cancer

**Figure 1:**
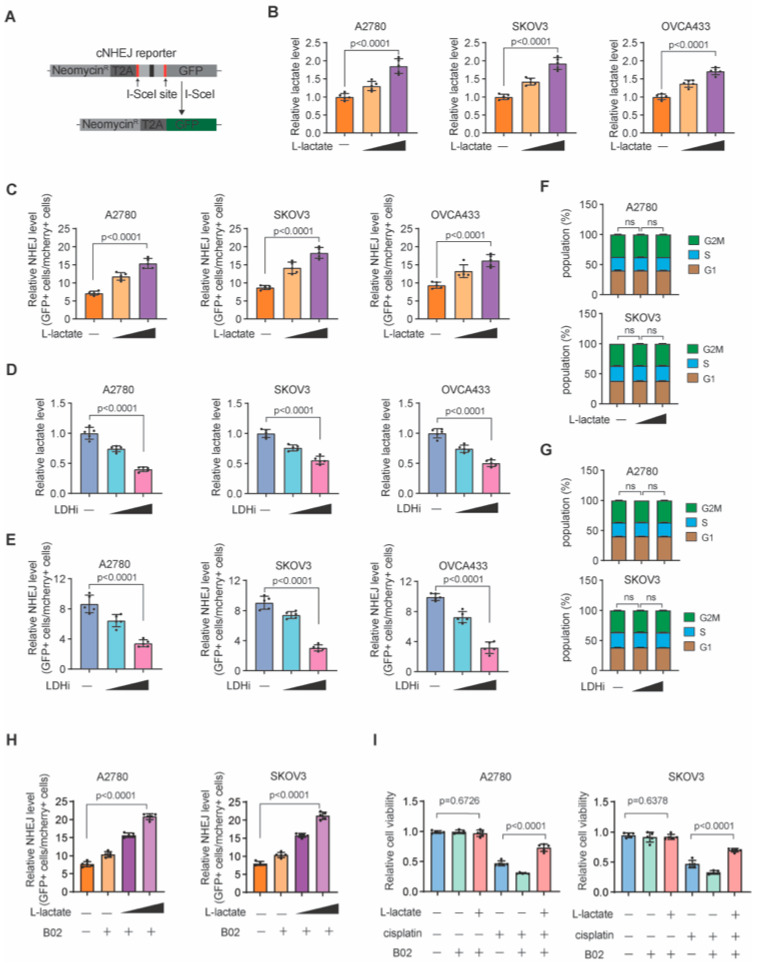
Lactate promotes NHEJ repair in ovarian cancer. (**A**) Illustration of cNHEJ reporter. Data are presented as the percentage of GFP-positive cells normalized to the percentage of mCherry-positive cells in each sample and are shown as mean ± SD from 3 biological replicates. (**B**) Relative lactate level in A2780, SKOV3, and OVCAR-433 ovarian cancer cell lines treated with increasing doses of L-lactate. (**C**) NHEJ level in A2780, SKOV3, and OVCAR-433 ovarian cancer cell lines treated with increasing doses of L-lactate. (**D**) Relative lactate level in A2780, SKOV3, and OVCAR-433 ovarian cancer cell lines treated with increasing doses of LDH inhibitor (LDHi). (**E**) NHEJ level in A2780, SKOV3, and OVCAR-433 ovarian cancer cell lines treated with increasing doses of LDHi. (**F**) Cell cycle distribution in A2780, SKOV3 ovarian cancer cell lines treated with increasing doses of L-lactate. (**G**) Cell cycle distribution in A2780, SKOV3 ovarian cancer cell lines treated with increasing doses of LDHi. (**H**) NHEJ level in A2780, SKOV3 ovarian cancer cell lines treated with increasing doses of L-lactate and B02. (**I**) Relative cell viability of A2780, SKOV3 ovarian cancer cell lines treated with L-lactate or B02 in the absence or presence of cisplatin. The vehicle drug treatment controls of L-lactate or LDHi were PBS or DMSO. Data are mean +/− SD from five biological replicates and analyzed with one-way ANOVA with Bonferroni’s post hoc test for (**B**–**E**,**H**,**I**). Exact *p*-values are provided in the Source Data. ns, not significant.

To investigate whether lactate could promote NHEJ repair in ovarian cancer, we first examined whether lactate is involved in the regulation of NHEJ by performing I-SecI-based NHEJ reporter assays (Figure 1A). A panel of ovarian cancer cell lines with the reporter plasmids transfected were treated with L-lactate. Consistent with previous reports, we confirmed that L-lactate resulted in marked increase in total cellular lactate levels (Figure 1B). We then examined these treated cells with the NHEJ reporter. As shown in Figure 1C, lactate treatment significantly increased NHEJ repair in all ovarian cancer examined, suggesting that protein lactylation promotes NHEJ repair. In cancer cells, lactate is mainly produced by lactate dehydrogenase (LDH) enzymes. We further treated the NHEJ reporter cells with LDH inhibitor (LDHi). As expected, LDHi treatment significantly decreased the lactate levels (Figure 1D). More importantly, we observed that NHEJ repair was markedly suppressed in reporter cells with LDH inhibited (Figure 1E). Notably, neither L-lactate nor LDHi treatment significantly altered cell cycle distribution, suggesting that the observed change in NHEJ repair efficiency was not caused by cell cycle change (Figure 1F,G). Furthermore, we utilized the HR inhibitor B02 to determine whether the cisplatin resistance conferred by lactate depends on a functional HR pathway. The results show that in the presence of the HR inhibitor B02, lactate treatment still significantly enhanced NHEJ repair efficiency and promoted cell survival under cisplatin treatment. (Figure 1H,I). Taken together, these data suggest that lactate may play a role in enhancing NHEJ repair in ovarian cancer cells.

### 3.2. Lactate Promotes Chemoresistance in Ovarian Cancer

**Figure 2:**
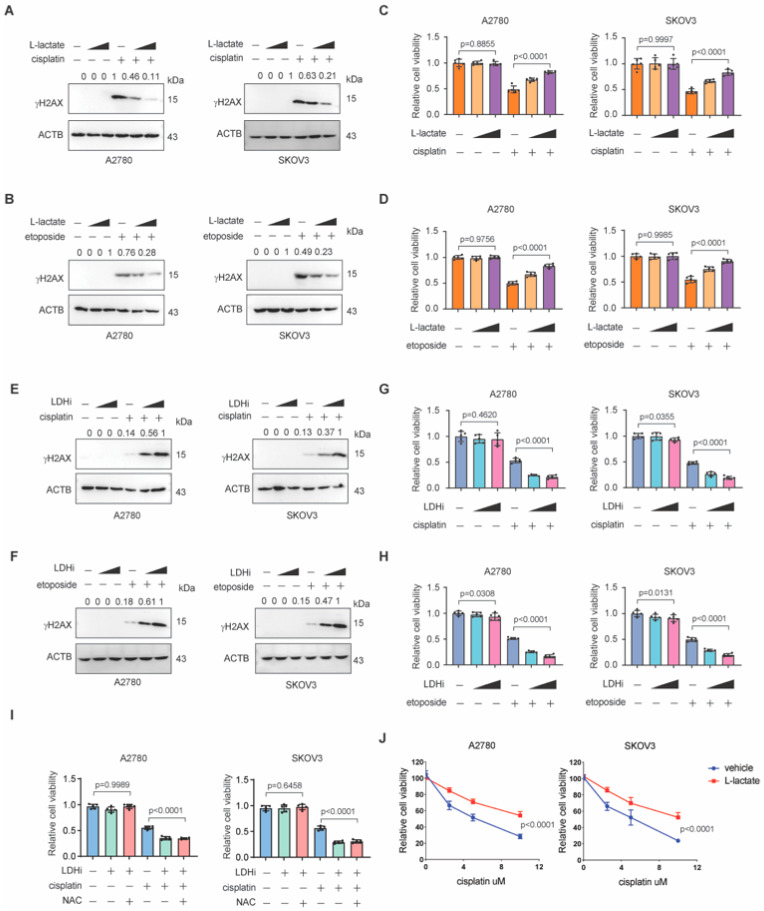
Lactate promotes chemoresistance in ovarian cancer. (**A**,**B**) Western blot of γH2AX level in ovarian cancer cell lines treated with increasing doses of L-lactate in the absence or presence of cisplatin (**A**) or etoposide (**B**). (**C**,**D**) Relative cell viability (% of untreated control) of A2780, SKOV3 ovarian cancer cell lines treated with increasing doses of L-lactate in the absence or presence of cisplatin (**C**) or etoposide (**D**). The vehicle drug treatment controls of L-lactate is PBS. (**E**,**F**) Western blot of γH2AX level in ovarian cancer cell lines treated with increasing doses of LDHi in the absence or presence of cisplatin (**E**) or etoposide (**F**). (**G**,**H**) Relative cell viability (% of untreated control) of A2780, SKOV3 ovarian cancer cell lines treated with increasing doses of LDHi in the absence or presence of cisplatin (**G**) or etoposide (**H**). (**I**) Relative cell viability (% of untreated control) of A2780, SKOV3 ovarian cancer cell lines treated with LDHi or NAC in the absence or presence of cisplatin. The vehicle drug treatment control of LDHi was DMSO. (**J**) CCK-8 assays conducted across multiple cisplatin concentrations (0, 2.5, 5, 10 µM) over 72 h. Data are representative of three independent biological experiments for (**A**,**B**,**E**,**F**). Data are representative of three independent biological experiments for (**A**,**B**,**E**,**F**). Data are mean +/− SD from five biological replicates and analyzed with one-way ANOVA with Bonferroni’s post hoc test for (**C**,**D**,**G**–**I**). Exact *p*-values are provided in the source data.

NHEJ repair is an important mechanism for cancer cells to alleviate DNA damage induced by chemotherapy and therefore can confer chemoresistance in cancer cells. Our observations that lactate could increase the NHEJ repair suggest that lactate might promote DNA damage repair and chemoresistance in ovarian cancer cells. To test this hypothesis, we treated ovarian cancer cells with lactate and evaluated the chemotherapy-induced DNA damage by examining the γH2AX level. As shown in Figure 2A,B, lactate treatment markedly decreased γH2AX level across the panels of ovarian cancer cells treated with cisplatin or etoposide, suggesting that lactate treatment facilitates DNA damage repair. Consistent with these observations, the cell viability assay showed that lactate treatment significantly increased resistance to chemotherapy in ovarian cancer cells (Figure 2C,D). In contrast, LDHi treatment strongly increased γH2AX level in ovarian cancer cells treated with cisplatin or etoposide, emphasizing the role of cellular lactate in supporting DNA damage repair (Figure 2E,F). In agreement, LDHi treatment significantly sensitized cancer cells to cisplatin or etoposide treatment (Figure 2G,H). In order to directly and decisively address whether lactate promotes chemoresistance primarily through modulating global redox balance, we performed a tailored NAC rescue experiment. Crucially, the addition of NAC failed to reverse this LDHi-induced chemosensitivity. Cell viability remained low and comparable to the LDHi treatment group alone (Figure 2I). Furthermore, we performed a comprehensive CCK-8 cell viability assays under longer treatment durations (72 h) and across a range of cisplatin concentrations to confirm the conclusion. As shown in Figure 2J, lactate supplementation significantly increased cell viability in a dose-dependent manner upon cisplatin exposure, confirming its protective role. (Figure 2J) Collectively, these data suggest that lactate promotes chemoresistance in ovarian cancer.

### 3.3. Lactate Promotes XRCC4–LIG4 Assembly to Facilitate NHEJ

**Figure 3:**
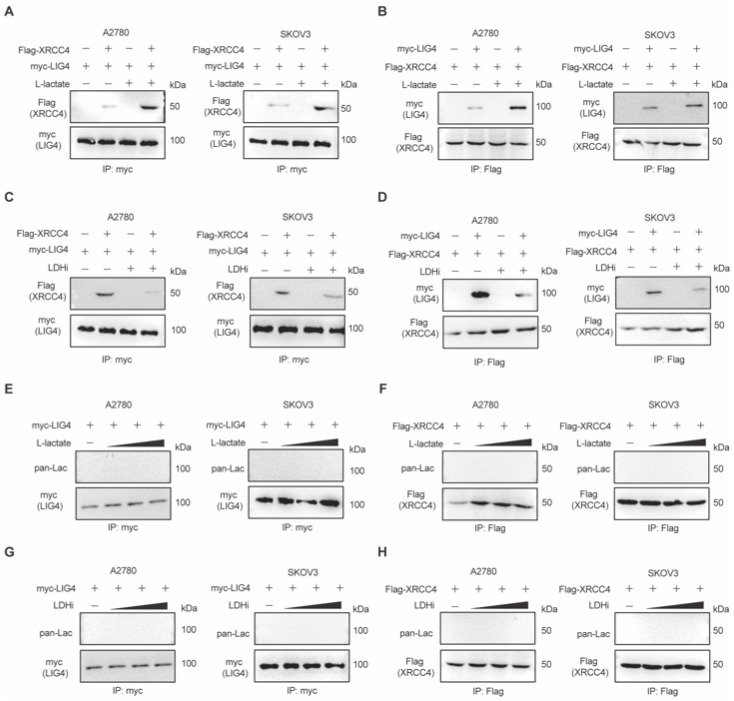
Lactate promotes XRCC4–LIG4 assembly to facilitate NHEJ. (**A**,**B**) Reverse immunoprecipitation of XRCC4 and LIG4 in ovarian cancer cell lines treated with L-lactate. (**C**,**D**) Reverse immunoprecipitation of XRCC4 and LIG4 in ovarian cancer cell lines treated with LDHi. (**E**,**F**) Lactylation of LIG4 (**E**) and XRCC4 (**F**) in ovarian cancer cell lines treated with L-lactate. (**G**,**H**) Lactylation of LIG4 (**G**) and XRCC4 (**H**) in ovarian cancer cell lines treated with LDHi. Data are representative of three independent biological experiments for all.

Next, we set out to explore how lactate could regulate NHEJ repair to confer chemoresistance. The DNA Ligase IV (LIG4)–XRCC4 complex is responsible for the ligation of broken DNA ends in the NHEJ pathway of DNA double strand break repair in mammals. XRCC4–LIG4 assembly plays a key role in NHEJ function. Therefore, we first investigated whether lactate may promote the XRCC4–LIG4 interaction. Indeed, the reciprocal immunoprecipitation of XRCC4 and LIG4 confirm that lactate markedly enhanced the interaction between XRCC4 and LIG4, with lactate treatment strongly promoting the interaction (Figure 3A,B), and LDHi treatment markedly suppressing the interaction (Figure 3C,D), respectively, in ovarian cancer cells. Previous studies have found that lactate could promote the MRN complex assembly through NBS1 lactylation [22]. Therefore, we first examined whether lactate could promote the lactylation of XRCC4 or LIG4. However, as shown in Figure 3E,F, no lactylation in XRCC4 or LIG4 was detected in ovarian cancer cells with or without lactate treatment. Similar results were obtained when cells were treated with or without LDHi (Figure 3G,H). These results suggest that lactate promotes XRCC4–LIG4 assembly to facilitate NHEJ independent of the lactylation of XRCC4 or LIG4.

### 3.4. Lactate Directly Binds to XRCC4 to Promote XRCC4–LIG4 Interaction

**Figure 4:**
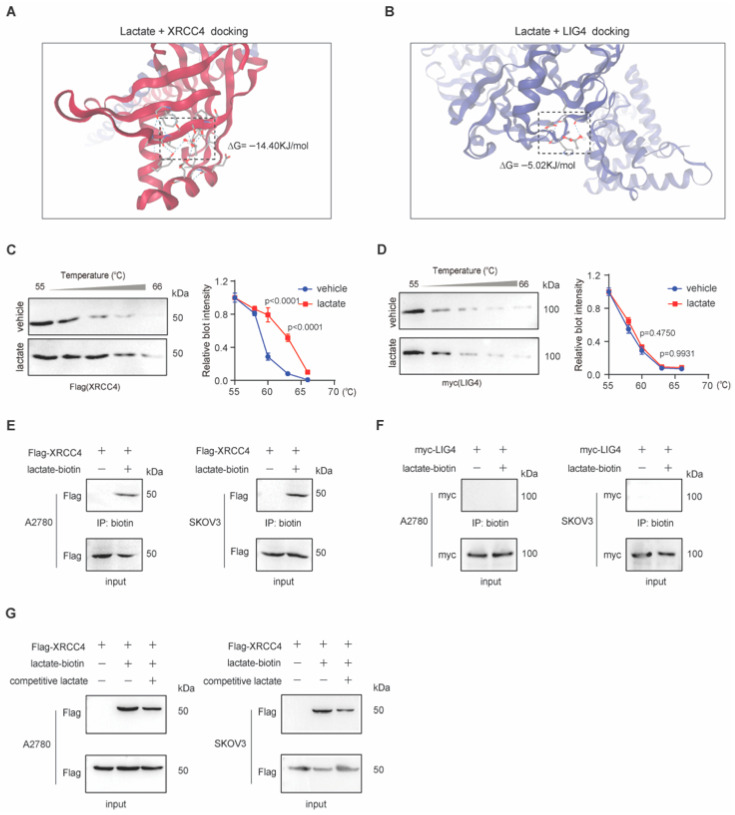
Lactate directly binds to XRCC4 to promote XRCC4–LIG4 interaction. (**A**,**B**) Molecular docking of L-lactate with XRCC4 (**A**) or LIG4 (**B**). (**C**,**D**) R Cellular thermal shift assay using cell lysate of Flag-XRCC4-overxpressing (**C**) or myc-LIG4-overexpressing (**D**) 293T cells. (**E**,**F**) Western blots of as indicated coIP and input samples. Lysate of cancer cells overexpressing Flag-XRCC4 (**E**) or my-LIG4 (**F**) was incubated with biotin-lactate before immunoprecipitation. The vehicle control for L-lactate treatment was PBS. (**G**) Western blots of as indicated coIP and input samples. Lysate of cancer cells overexpressing Flag-XRCC4 was incubated with biotin-lactate before immunoprecipitation in the absence or presence of a 40-fold excess of free (unlabeled) L-lactate as a competitor. Data are representative of three independent biological experiments for (**C**–**G**).

We next set to further explore how lactate could promote XRCC4–LIG4 assembly. Considering lactate did not regulate XRCC4 or LIG4 lactylation, we speculated whether lactate could directly bind to XRCC4 or LIG4. To test this hypothesis, we performed molecular docking. As shown in Figure 4A,B, lactate binds to XRCC4 with high affinity (ΔG), in contrast the binding between lactate and LIG4 is weak (ΔG), suggesting that lactate might directly bind to XRCC4. Supporting this, the cellular thermal shift assay also demonstrates that lactate directly binds to XRCC4; in contrast, the stability of LIG4 was not affected by the incubation of lactate (Figure 4C,D). Using the lactate-biotin probe, we further demonstrate that lactate directly binds to XRCC4 through biotin streptavidin immunoprecipitation. The cell lysate of ovarian cancer cells was incubated with lactate-biotin probe for streptavidin-conjugated magnetic beads immunoprecipitation. As shown in Figure 4E, using the lactate-biotin probe, we further demonstrate that lactate directly binds to XRCC4 through biotin streptavidin immunoprecipitation. The binding was specific, as it could be competed away by excess free lactate (Figure 4G). As shown in Figure 4F, XRCC4 but not LIG4 was immunoprecipitated using the lactate-biotin probe. Based on the above data, we conclude that lactate may directly bind to XRCC4 to promote XRCC4–LIG4 assembly.

### 3.5. Blocking Lactate-XRCC4 Binding Suppressed Lactate-Induced XRCC4–LIG4 Assembly and NHEJ Repair

**Figure 5:**
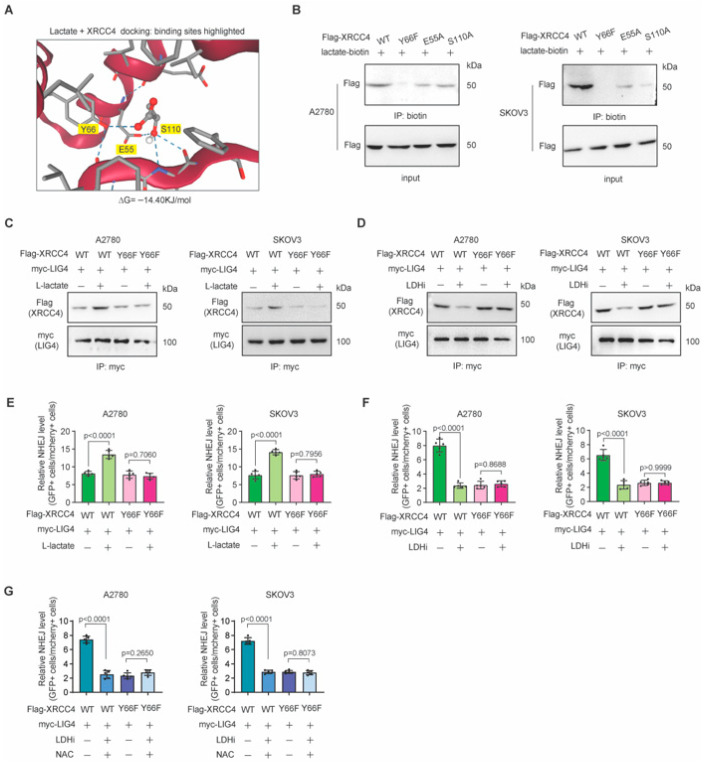
Blocking lactate-XRCC4 binding suppressed lactate-induced XRCC4–LIG4 assembly and NHEJ repair. (**A**) Molecular docking of L-lactate with XRCC4 with binding sites highlighted. (**B**) Western blots of as indicated coIP and input samples. Lysate of cancer cells overexpressing Flag-XRCC4 wildtype (WT), Y66F, E55A, or S110A mutant was incubated with lactate-biotin before immunoprecipitation. (**C**) Interaction of XRCC4 WT or XRCC4 Y66F mutant with LIG4 in cancers treated with L-lactate. (**D**) Interaction of XRCC4 WT or XRCC4 Y66F mutant with LIG4 in cancers treated with LDHi. (**E**) Relative NHEJ level in cancer cells overexpressing XRCC4 WT or XRCC4 Y66F mutant with LIG4 upon the treatment of L-lactate. (**F**) Relative NHEJ level in cancer cells overexpressing XRCC4 WT or XRCC4 Y66F mutant with LIG4 upon the treatment of LDHi. (**G**) Relative NHEJ level in cancer cells overexpressing XRCC4 WT or XRCC4 Y66F mutant with LIG4 upon the treatment of LDHi or NAC. Data are presented as the percentage of GFP-positive cells normalized to the percentage of mCherry-positive cells in each sample and are representative of three independent biological experiments for (**B**–**D**). Data are mean +/− SD from five biological replicates and analyzed with one-way ANOVA with Bonferroni’s post hoc test for (**E**–**G**). Exact *p*-values are provided in the source data.

To further explore whether lactate–XRCC4 binding is important for XRCC4–LIG4 assembly and NHEJ repair, we individually examined the potential lactate-binding sites including Y66, E55, and S110 of XRCC4, as shown by the molecular docking (Figure 5A). Indeed, we found that mutation of these sites significantly inhibited the binding of lactate-XRCC4, with Y66F mutation almost abolishing the binding, as shown by lactate-biotin-based immunoprecipitation (Figure 5B). Therefore, we chose the XRCC4 wild type and Y66F mutant for further study. We overexpressed the Flag-tagged XRCC4 wild type and Y66F mutant in endogenous XRCC4-depleted ovarian cancer NHEJ reporter cells. As shown in Figure 5C, while lactate treatment strongly promoted XRCC4–LIG4 interactions, it exhibited no effect on XRCC4–LIG4 interactions with XRCC4 Y66F mutated. Similar results were also observed when LDHi was applied in these cells, LDHi treatment also exhibited no effect on XRCC4–LIG4 interactions with XRCC4 Y66F mutated (Figure 5D).

Consistently, XRCC4 Y66F mutation almost completely abrogated the increase in NHEJ repair conferred by lactate treatment (Figure 5E). In agreement, XRCC4 Y66F mutation almost completely abrogated the inhibition in NHEJ repair conferred by LDHi (Figure 5F). To directly and definitively answer whether rescuing redox imbalance can affect the XRCC4–LIG4 interaction independently of lactate binding, we also performed a critical genetic rescue experiment. As shown in the figures, the addition of NAC failed to rescue the NHEJ efficiency in the XRCC4-WT/Y66F cells treated with LDHi (Figure 5G). Collectively, these data suggest that blocking lactate-XRCC4 binding by XRCC4 Y66F mutation suppressed lactate-induced XRCC4–LIG4 assembly and NHEJ repair, emphasizing the role of lactate in regulating XRCC4–LIG4 assembly and NHEJ repair.

### 3.6. Lactate-Mediated NHEJ Repair Confers Chemoresistance in Ovarian Cancer In Vivo

**Figure 6:**
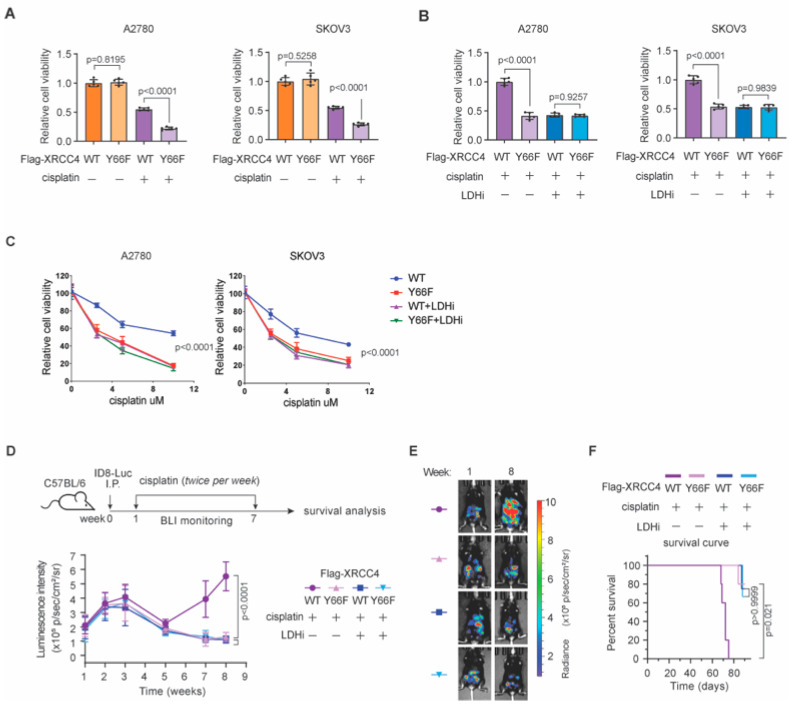
Lactate-mediated NHEJ repair confers chemotherapy resistance in ovarian cancer in vivo. (**A**) Relative cell viability of XRCC4-depleted cancer cells overexpressing XRCC4 WT or XRCC4 Y66F mutant in the absence or presence of cisplatin. (**B**) Relative cell viability of XRCC4-depleted cancer cells overexpressing XRCC4 WT or XRCC4 Y66F mutant in the absence or presence of LDHi upon cisplatin treatment. (**C**) CCK-8 assay of XRCC4-depleted cancer cells overexpressing XRCC4 WT or XRCC4 Y66F mutant in multiple cisplatin concentrations (0, 2.5, 5, 10 µM) over 72 h. (**D**,**E**) Illustration of in vivo mouse model work scheme and tumor growth determined by bioluminescence of XRCC4-depleted cancer cells overexpressing XRCC4 WT or XRCC4 Y66F mutant in the absence or presence of LDHi upon cisplatin treatment. E, representative BLI image. N = 5, two-way ANOVA. (**F**) Survival cure of mice in (**D**), N = 5, log-rank (Mantel-Cox) test. Data are mean +/− SD from five biological replicates and analyzed with one-way ANOVA with Bonferroni’s post hoc test for A and B. For panel E, data are mean ± SEM (N = 5 mice per group) and analyzed with two-way ANOVA with the Bonferroni’s post hoc test. For panel F, survival was compared using the log-rank (Mantel-Cox) test. Exact *p*-values are provided in the Source Data.

To test whether lactate-mediated NHEJ repair played a key role in DNA damage repair and chemoresistance, we compared chemotherapy response in XRCC4-depleted ovarian cancer cells with Flag-tagged XRCC4 wild type and Y66F mutant overexpressed. As shown in Figure 6A, while no differences in cell viability were found between XRCC4 WT or Y66F mutant, the XRCC4 Y66F mutant-expressing cells exhibited significantly higher sensitivity to chemotherapy compared to the XRCC4 WT cells (Figure 6A). More importantly, the difference between the XRCC4 WT and XRCC4 Y66F mutant in chemotherapy response were abolished when LDHi were applied (Figure 6B), suggesting that lactate-mediated NHEJ repair confers chemotherapy resistance. CCK-8 assay of XRCC4-depleted cancer cells overexpressing XRCC4 WT or XRCC4 Y66F mutant in multiple cisplatin concentrations (0, 2.5, 5, 10 µM) over 72 h further confirm that lactate-mediated NHEJ activation through XRCC4–LIG4 complex assembly is a key mechanism underlying chemoresistance (Figure 6C).

We further tested this idea in vivo using ID8-luciferase ovarian cancer model, especially considering the XRCC4 Y66F is conserved between human and mice. Mice transplanted with XRCC4 WT or Y66F mutant ovarian cancer cells were treated with cisplatin +/− LDHi. Compared with XRCC4 Y66F mutant, XRCC4 WT tumor showed resistance to chemotherapy treatment; in contrast, LDHi treatment significantly diminished the differential response to chemotherapy (Figure 6D–F). Taken together, these data suggest that lactate-mediated NHEJ repair confers chemotherapy resistance in ovarian cancer in vivo.

## 4. Discussion

In this study, we demonstrate that lactate enhances non-homologous end joining (NHEJ) repair and chemoresistance in ovarian cancer by facilitating the assembly of the XRCC4–LIG4 complex. Using an I-SceI-based NHEJ reporter assay, we observed that lactate significantly promoted NHEJ activity in ovarian cancer cell lines—a finding further validated through lactate supplementation and LDH inhibition experiments. Given that NHEJ is a critical mechanism for repairing chemotherapy-induced DNA damage, its upregulation by lactate likely contributes to chemoresistance. Consistent with this, we found that lactate treatment reduces γH2AX levels (a marker of DNA damage) and enhances cell viability under cisplatin exposure. The XRCC4–LIG4 complex plays a central role in NHEJ-mediated ligation of DNA double-strand breaks (DSBs) in mammalian cells. Our data reveal that lactate strengthens the interaction between XRCC4 and LIG4, thereby boosting NHEJ efficiency. Notably, while lactate does not induce the lactylation of XRCC4 or LIG4, it directly binds XRCC4 at Y66, promoting XRCC4–LIG4 complex formation. Disrupting this interaction via an XRCC4 Y66 mutation abolishes lactate-induced NHEJ activation and sensitizes ovarian cancer cells to cisplatin in vivo. From a clinical perspective, our findings underscore the therapeutic potential of targeting lactate metabolism or the lactate–XRCC4 interaction to overcome chemoresistance in ovarian cancer. Preclinical models demonstrate that lactate-driven NHEJ activation confers resistance to cisplatin, suggesting that inhibiting lactate production (e.g., with LDH inhibitors) or specifically disrupting the lactate–XRCC4 binding interface could restore chemosensitivity. These results establish lactate as a key metabolic regulator of NHEJ and highlight its role in cisplatin resistance.

Cancer metabolism has emerged as a critical determinant of chemoresistance, with accumulating evidence linking metabolic reprogramming to therapeutic failure [26,27]. Previous studies, including our own, have elucidated various metabolic pathways contributing to cisplatin resistance in ovarian cancer. For instance, we demonstrated that diacylglycerol kinase alpha (DGKA) promotes chemoresistance via its metabolic product, phosphatidic acid (PA), which activates the transcription factor c-JUN and upregulates the cell-cycle regulator WEE1 [28]. Additionally, inositol 1,3,4,5-tetrakisphosphate (IP4), generated by ITPKB, mitigates cisplatin-induced oxidative stress by suppressing NADPH oxidase 4 (NOX4), thereby supporting tumor survival [29]. More recently, we reported that tyrosine catabolism suppresses translesion DNA synthesis, sensitizing ovarian cancer cells to genotoxic chemotherapy [7]. The Warburg effect, a hallmark of cancer metabolism, leads to excessive lactate production, which has been implicated in chemoresistance primarily through its regulation of homologous recombination (HR) repair. However, the mechanisms by which lactate influences other DNA repair pathways, particularly NHEJ, remain poorly understood. Our findings bridge this gap by revealing lactate’s direct role in modulating NHEJ via XRCC4–LIG4 stabilization. Interestingly, a very recent study also confirmed that lactate could promote NHEJ, which found that glycolysis-derived lactate promotes XLF lactylation at K288 within its Ku-binding motif (X-KBM) to regulate NHEJ. Lactylation of XLF promotes NHEJ repair and chemoresistance in cancer [30]. Therefore, it seems that NHEJ could be regulated by lactate in both lactylation-dependent (e.g., via XLF) and lactylation-independent (e.g., via direct allosteric modulation of XRCC4, as shown here) manners, highlighting the multifaceted role of this metabolite in fine-tuning DNA repair pathways. Therefore, it seems that NHEJ could be regulated by lactate in both lactylation-dependent and lactylation-independent manners.

DNA double-strand breaks (DSBs) are predominantly repaired via two major pathways: NHEJ and homologous recombination (HR). NHEJ operates throughout the cell cycle, rapidly ligating broken DNA ends without requiring a homologous template, albeit with lower fidelity. In contrast, HR is restricted to the S and G2 phases, relying on sister chromatids for error-free repair. The XRCC4–LIG4 complex is indispensable for NHEJ, mediating the final ligation step of DSB repair [31]. Despite its importance, the regulatory mechanisms governing LIG4 activity and its interplay with other NHEJ factors remain incompletely understood. While prior studies have established that HR is metabolically regulated—for example, through MRE11 and NBS1 lactylation—whether NHEJ is similarly influenced by metabolic cues was unknown. Our work provides the first evidence that lactate directly enhances NHEJ by binding XRCC4 at Y66, thereby stabilizing the XRCC4–LIG4 interaction. Future structural studies should elucidate the atomic details of the XRCC4/lactate complex, which may offer insights into how lactate modulates NHEJ efficiency.

From a clinical perspective, our findings underscore the therapeutic potential of targeting lactate metabolism to overcome chemoresistance in ovarian cancer. Preclinical models demonstrate that lactate-driven NHEJ activation confers resistance to cisplatin, suggesting that inhibiting lactate production or disrupting lactate–XRCC4 binding could restore chemosensitivity. Moreover, our results may explain why HR-deficient (HRD) tumors eventually develop resistance to PARP inhibitors and other genotoxic agents: compensatory NHEJ hyperactivation, fueled by lactate, could serve as an escape mechanism [32]. Moving forward, the development of specific inhibitors targeting lactate synthesis or its interaction with XRCC4 may provide a novel strategy to circumvent chemoresistance and improve patient outcomes.

## Data Availability

Source data in this study are available in the Supplementary Materials.

## Supplementary Materials

The following supporting information can be downloaded at: https://www.mdpi.com/article/10.3390/biomedicines13122949/s1, Table S1: key reagents information; Source data.
