# Supplementary material for: Lactate Enhances Non-Homologous End Joining Repair and Chemoresistance Through Facilitating XRCC4–LIG4 Complex Assembly in Ovarian Cancer
Source: Biomedicines. 2025 Nov 30;13(12):2949. doi: 10.3390/biomedicines13122949 (PMC12730564; doi:10.3390/biomedicines13122949)
Supplement: Supplementary file 1 [file biomedicines-13-02949-s001.zip › Manuscript-supplementary/Supplementary table S1.Key reagents information.pdf]

KEY RESOURCES TABLE

REAGENT or RESOURCE	SOURCE	IDENTIFIER
Antibodies		
γH2AX(for WB 1:1000 dilutions)	Cell Signaling Technology	Cat#80312, RRID: AB_2799949
ACTB (for WB 1:2500 dilutions)	Sigma–Aldrich	Cat#A1978, RRID: AB_476692
Flag (mouse) (for WB 1:1000 dilutions)	Sigma–Aldrich	Cat#F1804, RRID: AB_262044
Flag (rabbit) (for WB 1:1000 dilutions)	Sigma–Aldrich	Cat#F7425, RRID: AB_439687
Myc-Tag (9B11) Mouse Antibody (for WB 1:1000 dilutions)	Cell Signaling Technology	Cat#2276T RRID:AB_331783
Anti-Pan-Lac (for WB 1:1000 dilutions)	PTM Biolab	Cat# PTM-1401RM
Bacterial and virus strains		
Stbl3	Thermo Fisher Scientific	Cat#C737303
Chemicals, peptides, and recombinant proteins		
cisplatin	Sigma–Aldrich	Cat#P4394, CAS: 15663-27-1
Etoposide	Sigma–Aldrich	Cat#341205,CAS：33419-42-0
L-lactic acid	Sigma-Aldrich	Cat# L1750
Lactate-Biotin	MCE	Cat# HY-B2227
NAC	MCE	Cat#HY-B0215
RAD51 Inhibitor B02	MCE	Cat#HY-101462
LDH inhibitor (Sodium oxamate)	Sigma-Aldrich	Cat# O2751
Polybrene	Sigma-Aldrich	Cat# H9268
Polyethylenimine (PEI)	Polysciences	Cat# 23966-100mg
DTT	Sigma-Aldrich	Cat# 3483-12-3
NP-40	Sigma-Aldrich	Cat# 127087-87-0
Agarose	Sigma-Aldrich	Cat#A9539, CAS: 9012-36-6
HEPES	Sigma-Aldrich	Cat# H3662
Lipofectamine™ 2000	Thermo Fisher Scientific	Cat# 11668027
Lipofectamine™ 3000	Thermo Fisher Scientific	Cat# L3000150

DAPI	Sigma–Aldrich	Cat#D9542, CAS: 28718-90-3
DMEM	Corning	Cat#10-013-CV
RPMI-1640	Corning	Cat#10-040-CV
McCoy's 5A	Thermo Fisher Scientific	Cat#16600082
DMEM/Ham's F-12 50/50	Corning	Cat#10-092-CV
FBS	Coring	Cat#35-081-CV
GlutaMax	Thermo Fisher Scientific	Cat#35050061
Penicillin–Streptomycin	Thermo Fisher Scientific	Cat#15140122
Insulin-Transferrin-Selenium-Ethanolamine	Thermo Fisher Scientific	Cat#51500056
Bovine Serum Albumin	Sigma-Aldrich	Cat# V900933
goat serum	Thermo Fisher Scientific	Cat#16210072
DMSO	Sigma-Aldrich	Cat#D6250
protease inhibitor cocktail	Roche	Cat# 04693116001
streptavidin magnetic beads	Cell Signaling Technology	Cat#5947
3x Flag peptide	Sigma-Aldrich	Cat#F4799
Puromycin	Sigma-Aldrich	Cat#540222, CAS: 58-58-2
T4 DNA Ligase	New England Biolads	Cat#M0202L
Q5® Hot Start High-Fidelity DNA Polymerase	New England Biolads	Cat# M0493L
EcoRI-HF®	New England Biolads	Cat# R3101L
AgeI-HF®	New England Biolads	Cat# R3552L
T4 Polynucleotide Kinase	New England Biolads	Cat#M0201L
XhoI	New England Biolads	Cat# R0146L
DPNI	New England Biolads	Cat# R0176L
nitrocellulose membranes	Bio-Rad	Cat#1620115
Critical commercial assays		
Protein Thermal Shift Dye Kit	Thermo Fisher Scientific	Cat#4461146

Trypan blue exclusion kit	Beyotime	Cat#C0011
pENTR™/D-TOPO™ clone kit	Thermo Fisher Scientific	Cat#K240020
QuikChange II XL Site-Directed Mutagenesis Kit	Agilent	Cat#200521
Gateway LR Clonase II Enzyme Mix	Thermo Fisher Scientific	Cat#1791-100
Anti-FLAG M2 Magnetic Beads	Sigma–Aldrich	Cat#M8823
Pierce Pull-Down Biotinylated Protein:Protein Interaction Kit	Thermo Fisher Scientific	Cat#21115
Myc-tag Protein IP Assay Kit with Magnetic Beads	Beyotime	Cat#P2183S
Plasmid Extraction Kit	TIANGEN	Cat# DP103
EndoFree Midi Plasmid Kit	TIANGEN	Cat# GDP108
EndoFree Maxi Plasmid Kit	TIANGEN	Cat# GDP117
CellTiter-Fluor Cell Viability Assay	Promga	Cat#G6080
Detergent Compatible Bradford Protein Assay Kit	Beyotime	Cat# P0006C
Experimental models: Cell lines		
Human: A2780	Sigma–Aldrich	Cat#93112519
Human: SKOV3	ATCC	Cat#HTB-77
Human: 293T	ATCC	Cat#CRL-3216
Mouse: ID8	Sigma–Aldrich	Cat#SCC145
Experimental models: Organisms/strains		
Mouse: C57BL/6NCrl	Charles Rivers	Cat#213, Vital River
Recombinant DNA		
pLCN DSB Repair Reporter (NHEJ reporter)	Arnoult et al.[25]	Addgene Plasmid #98895
pLHCX-mCherry	This study	N/A
pLS-SceI	a gift from Nadav Ahituv	Addgene Plasmid #137725
pLHCX: Flag-XRCC4 M1	This study	N/A
pLHCX: Flag-XRCC4 M2	This study	N/A
pLHCX: Flag-XRCC4 M3	This study	N/A
pLHCX: Flag-XRCC4 (wild type)	This study	N/A
pLX304: myc-LIG4	This study	N/A
Software and algorithms		

FlowJo (v10.0.7)	Treestar	https://www.flowjo.com/
ImageJ	NIH	https://imagej.net/software/fiji/
Prism (v8.2)	GraphPad	https://www.graphpad.com/
