# Supplementary material for: Lactate Enhances Non-Homologous End Joining Repair and Chemoresistance Through Facilitating XRCC4–LIG4 Complex Assembly in Ovarian Cancer
Source: Biomedicines. 2025 Nov 30;13(12):2949. doi: 10.3390/biomedicines13122949 (PMC12730564; doi:10.3390/biomedicines13122949)
Supplement: Supplementary file 1 [file biomedicines-13-02949-s001.zip › Manuscript-supplementary/source data.zip!/source data/uncropped gel.pdf]

Figure 2

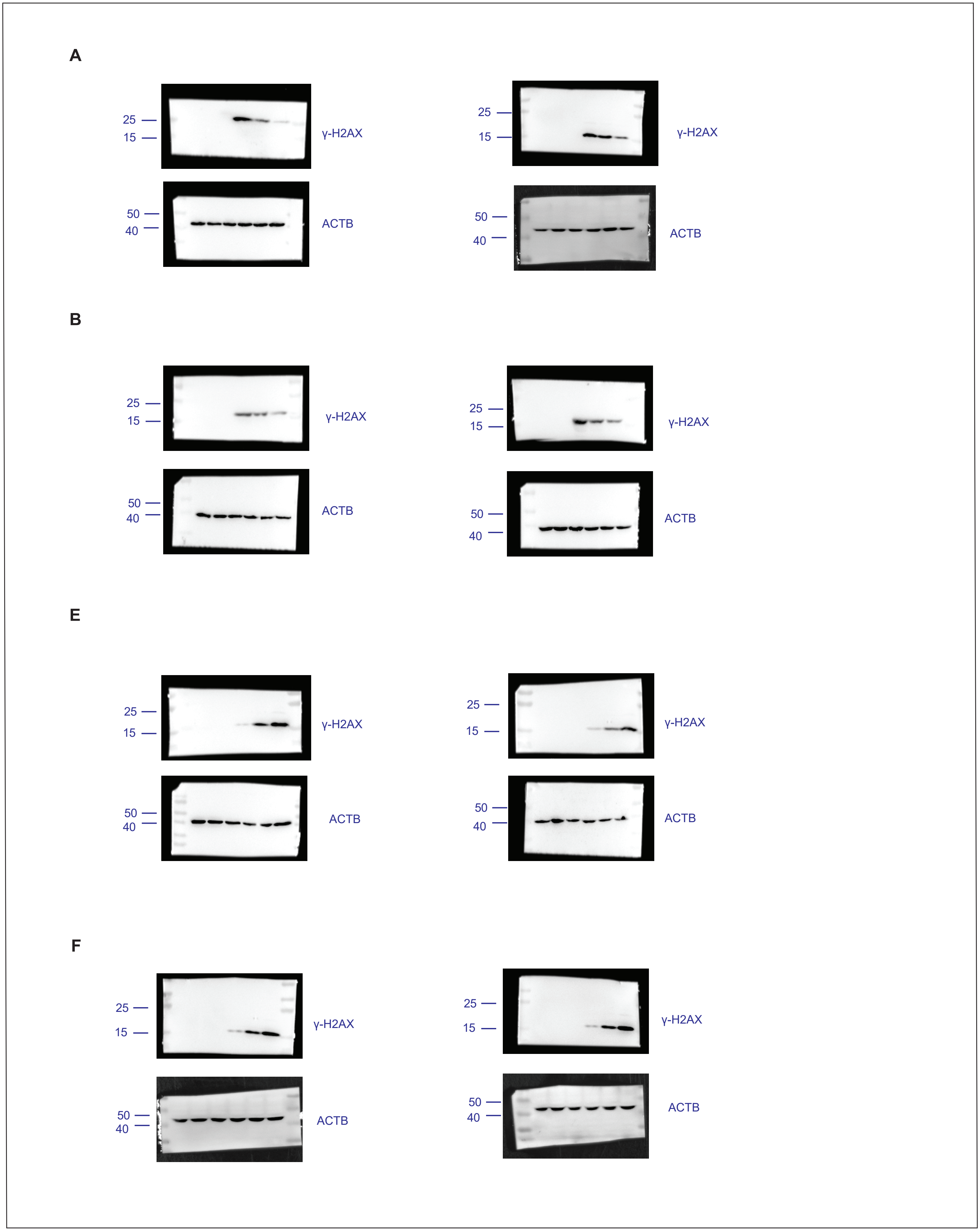

Figure 3

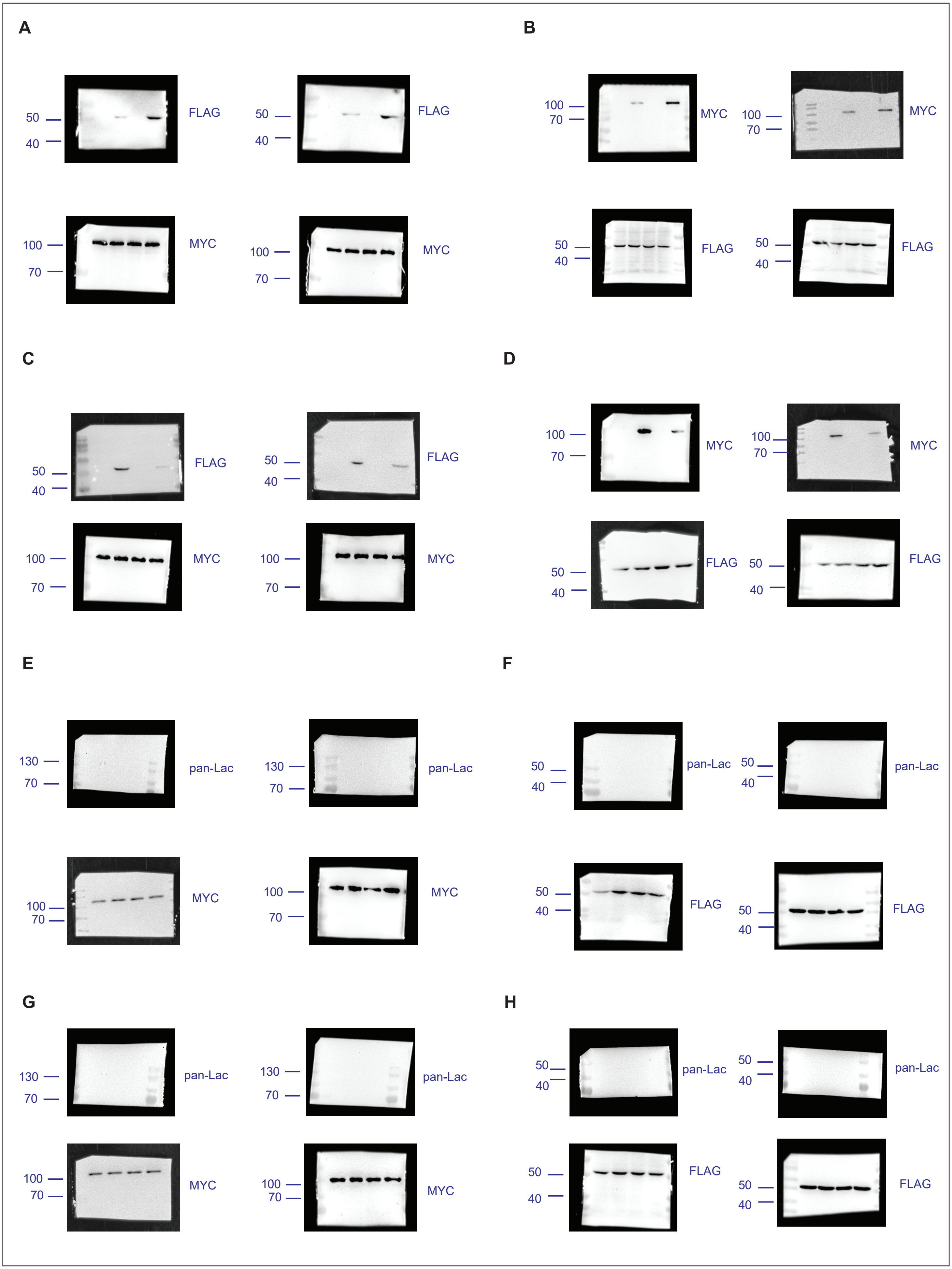

A
50
40
FLAG
100
70
MYC
B
100
70
MYC
50
40
FLAG
C
50
40
FLAG
100
70
MYC
D
100
70
MYC
50
40
FLAG
E
130
70
pan-Lac
100
70
MYC
F
50
40
pan-Lac
50
40
FLAG
G
130
70
pan-Lac
100
70
MYC
H
50
40
pan-Lac
50
40
FLAG

Figure 4

C

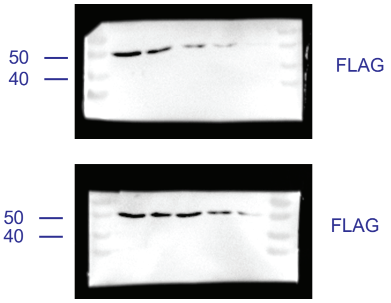

D

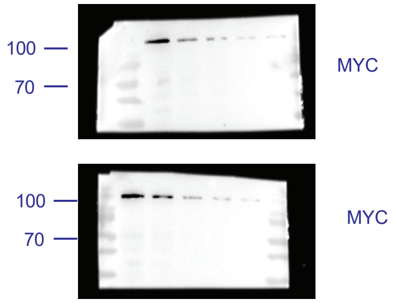

E

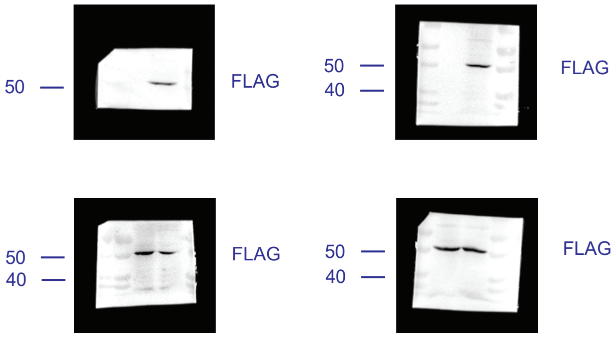

F

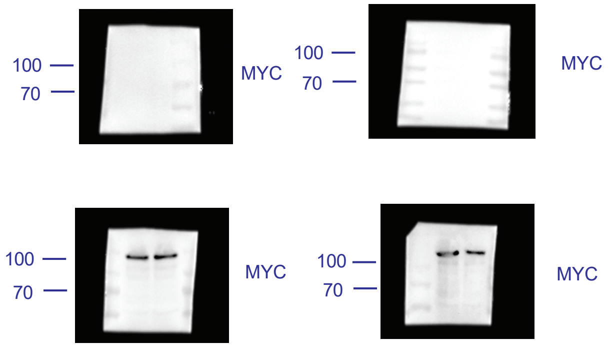

G

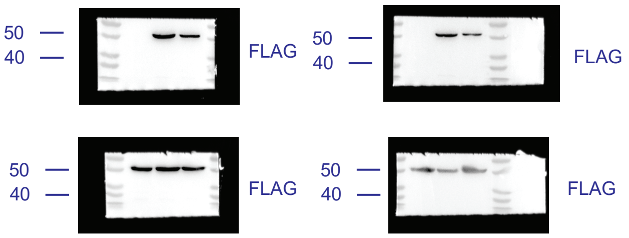

Figure 5

B

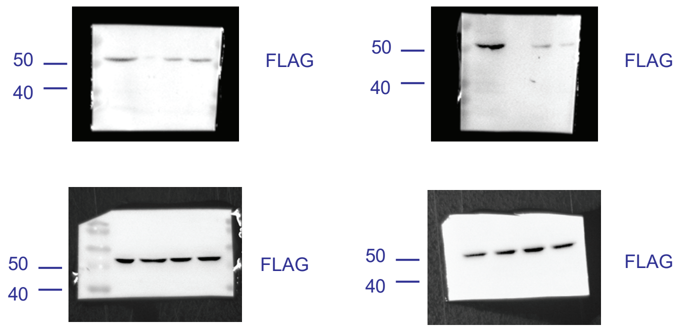
50
40
FLAG
50
40
FLAG
50
40
FLAG
50
40
FLAG

C

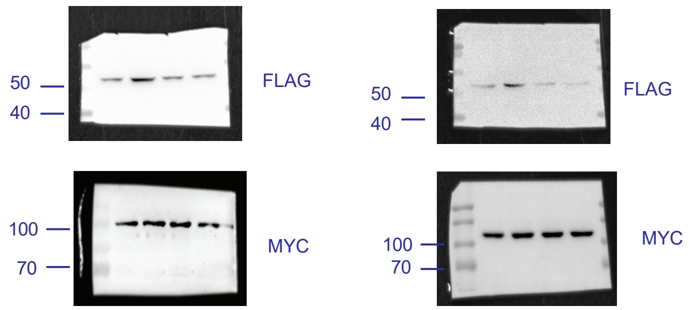
50
40
FLAG
50
40
FLAG
100
70
MYC
100
70
MYC

D

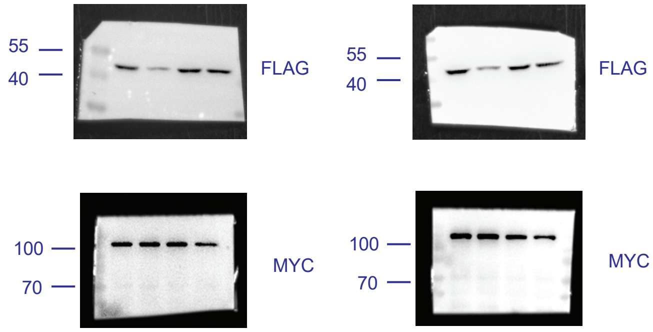
55
40
FLAG
55
40
FLAG
100
70
MYC
100
70
MYC
